# Optimal tentative abdominal closure for open abdomen: a multicenter retrospective observational study (OPTITAC study)

**DOI:** 10.1097/JS9.0000000000000687

**Published:** 2023-09-02

**Authors:** Ryo Yamamoto, Shunsuke Kuramoto, Masayuki Shimizu, Hiroharu Shinozaki, Tasuku Miyake, Yoshihiko Sadakari, Kazuhiko Sekine, Yasushi Kaneko, Ryo Kurosaki, Kiyoshi Koizumi, Takayuki Shibusawa, Yoshihiko Sakurai, Sota Wakahara, Junichi Sasaki

**Affiliations:** a Department of Emergency and Critical Care Medicine, Keio University School of Medicine, Tokyo; b Department of Emergency and Critical Care Medicine, Tokyo Saiseikai Central Hospital, Tokyo; c Department of Emergency and Critical Care Medicine, National Hospital Organization Tokyo Medical Center, Tokyo; d Department of Emergency and Critical Care Medicine, Keio University School of Medicine, Tokyo; e Department of Surgery, St Mary’s Hospital, Fukuoka; f Department of Surgery, Kenwakai Otemachi hospital, Fukuoka; g Department of Acute Care Surgery, Shimane University Faculty of Medicine, Shimane; h Department of Surgery, Japanese Red Cross Maebashi Hospital, Gunma; i Department of Cardiovascular Surgery, Ashikaga Red Cross Hospital, Tochigi; j Department of Surgery, Saiseikai Utsunomiya Hospital, Tochigi; k Department of Surgery, Shonantobe General Hospital, Kanagawa; l Department of Trauma and Emergency Surgery, Saiseikai Yokohamashi Tobu Hospital, Kanagawa; m Department of Emergency Medicine, Hiratsuka City Hospital, Kanagawa; n ryoyamamoto@keio.jp

**Keywords:** abdominal sepsis, damage control surgery, fascia closure, high negative pressure, negative-pressure wound therapy

## Abstract

**Background:**

Primary fascia closure is often difficult following an open abdomen (OA). While negative-pressure wound therapy (NPWT) is recommended to enhance successful primary fascia closure, the optimal methods and degree of negative pressure remain unclear. This study aimed to elucidate optimal methods of NPWT as a tentative abdominal closure for OA to achieve primary abdominal fascia closure.

**Materials and Methods:**

A multicenter, retrospective, cohort study of adults who survived OA greater than 48 h was conducted in 12 institutions between 2010 and 2022. The achievement of primary fascia closure and incidence of enteroatmospheric fistula were examined based on methods (homemade, superficial NPWT kit, or open-abdomen kit) or degrees of negative pressure (<50, 50–100, or >100 mmHg). A generalized estimating equation was used to adjust for age, BMI, comorbidities, etiology for laparotomy requiring OA, vital signs, transfusion, severity of critical illness, and institutional characteristics.

**Results:**

Of the 279 included patients, 252 achieved primary fascia closure. A higher degree of negative pressure (>100 mmHg) was associated with fewer primary fascia closures than less than 50 mmHg [OR, 0.18 (95% CI: 0.50–0.69), *P*=0.012] and with more frequent enteroatmospheric fistula [OR, 13.83 (95% CI: 2.30–82.93)]. The methods of NPWT were not associated with successful primary fascia closure. However, the use of the open-abdomen kit was related to a lower incidence of enteroatmospheric fistula [OR, 0.02 (95% CI: 0.00–0.50)].

**Conclusion:**

High negative pressure (>100 mmHg) should be avoided in NPWT during tentative abdominal closure for OA.

## Introduction

Highlights

- A multicenter, retrospective, cohort study on negative-pressure wound therapy during tentative abdominal closure for an open abdomen was conducted.
- Negative pressure less than −100 mmHg was associated with fewer primary fascia closures and more frequent enteroatmospheric fistula.
- Any methods of negative-pressure wound therapy were not associated with successful primary fascia closure.

The open abdomen (OA) procedure is widely used for laparotomy without abdominal fascia closure in patients with various conditions, including intra-abdominal organ injury, abdominal compartment syndrome, and abdominal sepsis^1–5^. OA enables sequential intra-abdominal lavage and reconstruction of the abdominal organs with stabilized hemodynamics. It improves the survival rates of trauma patients who undergo damage control surgery^2,6^. However, a considerable number of patients with OA experienced difficulty in achieving primary abdominal fascia closure, which prolongs hospital stay and causes chronic abdominal pain and severe nutritional disturbance^7,8^.

In addition to intensive care with meticulous fluid management, several surgical techniques have been developed to achieve primary fascia closure following OA^1,8–12^. Negative-pressure wound therapy (NPWT)^13^, dynamic closure technique with traction devices^14^, component separation^15^, and local or free flap are typical options^8^. While many have a successful rate of primary fascia closure^11^, NPWT is the most frequently used because it can effectively remove intra-abdominal fluid and prevent the retraction of the incisional wound edges^10,16^, which enhances primary fascia closure. Some clinical management guidelines recommend the use of NPWT during tentative abdominal closure for OA, regardless of the reason for leaving the abdomen open^1,9^.

NPWT can be applied with a commercially available NPWT system or a hospital developed system, using resources such as a suction pump, tubing, surgical gauze, and plastic surgical dressing^17^. An NPWT system designed for OA is also commercially available, but the optimal method of NPWT for tentative abdominal closure has not been sufficiently validated^1^. As the appropriate degree of negative pressure remains undetermined, it is usually decided by the treating physician or institutional policy^9^. While higher negative pressure greater than 80 mmHg has been reported to increase intestinal injury and up to 120 mmHg of negative pressure leads to greater fluid drainage^9,18^, the optimal range of negative pressure remains unclear.

This multicenter retrospective cohort study investigated patients who underwent OA and examined the methods of tentative abdominal closure and their clinical consequences. This study aimed to elucidate the optimal NPWT for tentative abdominal closure in OA to achieve primary fascia closure. We hypothesized that a specific method or range of negative pressure would be associated with a higher rate of primary abdominal fascia closure following OA.

## Material and methods

### Study design and setting

This was a nationwide multicenter retrospective cohort study conducted by the Optimal Tentative Abdominal Closure for Open Abdomen (OPTITAC) study group from January 2010 to March 2022. The OPTITAC study included patients who underwent OA at 12 participating tertiary care centers. The study was registered at the University Hospital Medical Information Network Clinical Trial Registry on 23 June 2021, prior to study initiation. The OPTITAC study was approved by the institutional review boards of all participating institutions. This study was conducted in accordance with the Helsinki Declaration. Written informed consent was waived due to the anonymity of the data. The current study was reported in line with the Strengthening the Reporting of Cohort Studies in Surgery (STROCSS) criteria^19^ (Supplemental Digital Content 1, http://links.lww.com/JS9/A944).

The OPTITAC study included patients aged 18 years or older who underwent OA with any etiology for laparotomy. OA was defined as performing a laparotomy without closing the abdominal fascia, regardless of the methods for tentative abdominal closure. Throughout the study period, OA was used for severe abdominal injury and abdominal compartment syndrome. OA as a treatment option for abdominal sepsis and nontraumatic intra-abdominal bleeding gradually spread across study institutions in the latter half of the study period. The decision for OA was made by the treating physician or following institutional policy. However, most patients with hemodynamic instability with any of the above indications underwent OA.

The methods and devices for tentative abdominal closure, including NPWT, varied depending on the preference of surgeons and resource availability. Commercially available NPWT could be used in all participating institutions, whereas NPWT devices specifically designed for OA were available from 2017. While the degree of negative pressure was determined by the treating surgeon without a uniform protocol for the current study, it was likely determined following institutional policy or preference. The time interval for definitive laparotomy, timing of reconstruction of abdominal organs, intensive care during OA, and procedures for fascia closure were also decided by the surgeons or institutions.

### Study population

Electronic data and relevant documentation were reviewed retrospectively at each institution. This study included OA patients who were 18 years or older. We excluded patients who died within 48 h of the initial laparotomy requiring OA, in whom resuscitation was withheld or withdrawn within 48 h of the initial laparotomy requiring OA, who had an abdominal incisional hernia impeding primary fascia closure, who did not undergo NPWT for tentative abdominal closure, or who had missing data on primary fascia closure.

### Data collection and definition

Patient data were retrospectively collected at each institution. The data included patient demographics, including age, sex, BMI, Charlson Comorbidity Index, past history of laparotomy and ventral hernia. It also included data regarding medications, such as anticoagulants, antiplatelets, and corticosteroids; details of the reason for OA; clinical information from before the laparotomy requiring OA, such as vital signs, laboratories, Sequential Organ Failure Assessment (SOFA) score, ventilator use, and resuscitation with fluid and transfusion; intraoperative information, including operation time, the amount of fluid and transfusion, and urine output; surgical methods for tentative abdominal closure, including methods and degree of negative pressure for NPWT; information from after the initial laparotomy, such as lactate value, fluid management, and transfusion; and information related to any sequential laparotomies were obtained. In addition, completion of primary fascia closure with the timing and methods; mortality at 28-day after the initiation of OA; ICU-, ventilator-, and renal replacement therapy (RRT)-free days up to 28 days, and adverse events after the initiation of OA were also retrieved.

The methods for NPWT were classified into homemade NPWT, a commercially available NPWT device (superficial NPWT kit), and a commercially available abdominal closure-specific NPWT device (open-abdomen kit). In homemade NPWT, a nonadhesive dressing was placed under the abdominal wall to cover the intra-abdominal organs, surgical gauze or a towel was packed in the incisional wound, plastic surgical dressing covered the whole would, and a suction system was attached to the dressing to keep negative pressure in the abdomen. The degree of negative pressure was classified into less than 50 mmHg, 50–100 mmHg, and greater than 100 mmHg, according to the pressure used at the initiation of OA. Detailed information regarding indications for each method or degree of negative pressure was not obtained for the study.

### Outcome measures

The primary outcome was the achievement of primary abdominal fascia closure, defined as the surgical approximation of the incision of the abdominal fascia at the end of surgery. Fascia closure after planned incisional hernia formation was not considered as primary fascia closure. Secondary outcomes included enteroatmospheric fistula and any other abdominal adverse events after the initiation of OA (anastomotic leakage, wound dehiscence, superficial and deep surgical site infection, and ventral hernia). All adverse events were diagnosed by the treating surgeons.

### Statistical analysis

Patient data were classified into primary fascia closure and no-fascia-closure groups. These were compared for patient characteristics, details of preoperative, intraoperative, and postoperative information, details for tentative abdominal closure for OA, and clinical consequences.

To elucidate the optimal method or range of negative pressure during OA, patients were divided depending on such exposures in the design of the retrospective cohort study. Before the analysis of the association between a method or degree of negative pressure for NPWT and the achievement of primary fascia closure, missing nonoutcome values were replaced with a set of substituted plausible values by creating five complete data sets using multiple imputation by the chained equation method^20^. Estimated associations in each of the imputed data sets were averaged to give an overall estimated associations. To adjust for potential confounders such as patient characteristics, preoperative information, and resuscitation content, multivariate analyses were conducted. As considerable differences between institutions were also expected regarding NPWT methods and the degree of negative pressure, a multivariable logistic regression model fitted with generalized estimating equations (GEE) was developed to adjust for patient populations and institutional differences^21^. In this analysis, a multilevel model was implemented; potential confounding factors related to patients and treatments were adjusted in multivariable logistic regression, and institutional differences were accounted by within‐institution clustering.

Relevant covariates were selected from known or possible predictors for achieving primary abdominal fascia closure in patients with OA^1,8,9,11,12,16,22,23^, including age, BMI, Charlson Comorbidity Index, chronic medication use (anticoagulants and antiplatelets), etiology for the laparotomy requiring OA (trauma vs. nontrauma), vital signs before the laparotomy (consciousness, systolic blood pressure [SBP], heart rate, and respiratory rate), and ventilator use, SOFA score, and transfusion before the laparotomy. Furthermore, to follow standard upper limits for the number of covariates in a multivariate logistic regression model (used as a link function in GEE), age, etiology of laparotomy, and SOFA score before laparotomy were entered into the primary GEE model, with the methods of NPWT and the degree of negative pressure. The relationship between a method or degree of negative pressure for NPWT and the achievement of primary fascia closure was then examined.

The secondary outcomes were also examined using GEE analyses with the same covariates as the primary model. The association between adverse event incidence and NPWT methods or the degree of negative pressure was analyzed.

Four sensitivity analyses were performed to examine the robustness of the primary results. First, a GEE analysis using all relevant covariates was conducted to maximally adjust patient background characteristics, accepting the overfitting of the model. Second, to balance the background adjustment and overfitting of the model, another GEE model was developed using BMI, comorbidities, ventilator use before surgery, and vital signs prior to laparotomy, along with the variables in the primary model. Third, to consider the difference in postoperative management after initial OA between physicians and institutions, another GEE model incorporated postoperative fluid balance within 24 h and consecutive NPWT use in following surgeries in addition to the original covariates in the primary analysis. Fourth, multivariate logistic regression analysis was performed with the same variables as the GEE model to avoid overestimating the effects of within‐institution clustering.

Subgroup analyses were performed to examine the association between the NPWT method or the degree of negative pressure, clinical characteristics, and primary fascia closure following OA. GEE analyses were repeated in patient subgroups determined by years when the OA was conducted (after 2017 when the open-abdomen kit became available), degree of intraoperative hemorrhage (≤500 ml and ≤1000 ml), and postoperative lactate value (≥4 mmol/l vs. <4 mmol/l). The same covariates as the primary GEE model were used.

Descriptive statistics are presented as median (interquartile range) or number (percentage). Considering the limited sample size, the differences in patient characteristics between those with and without primary fascia closure were shown with a standardized difference, in which a standardized difference greater than 0.3 was considered substantial^24^. The hypothesis was tested only on the primary outcome in which an α error rate of 0.05 was considered statistically significant in a two-sided test. The secondary outcomes are shown with an odds ratio (OR) and 95% CI. All statistical analyses were performed using IBM SPSS Statistics for Windows version 28.0 (IBM Corp.).

## Results

### Patient characteristics

**Figure 1:**
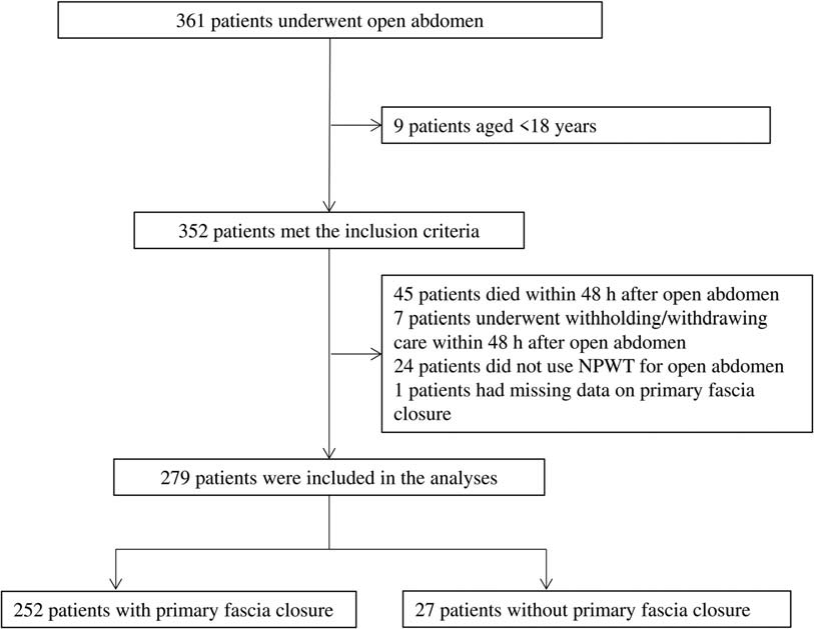
Patient flow diagram. Of 361 patients who underwent OA in the study period, 279 adult patients survived for greater than 48 h and underwent NPWT for tentative abdominal closure during OA; therefore, they were eligible for this study. Of these patients, 252 (90.3%) achieved primary abdominal fascia closure. OA, open abdomen; NPWT, negative pressure wound therapy.

Of 361 patients who underwent OA in the study period, 279 adult patients survived for greater than 48 h and underwent NPWT for tentative abdominal closure during OA; therefore, they were eligible for this study (Fig. 1). Among them, 252 (90.3%) patients achieved primary abdominal fascia closure.

Table 1 shows the patient’s characteristics. Patients with primary fascia closure had a lower BMI and preoperative SOFA score; a higher Charlson Comorbidity Index, SBP, and albumin before laparotomy, and received fewer transfusions before and during the initial surgery than those with no-fascia-closure. In addition, fewer patients with primary fascia closure had a history of laparotomy and ventral hernia and underwent OA due to abdominal compartment syndrome rather than abdominal trauma.

**Table 1:** Characteristics of patients with open abdomen.

	Primary fascia closure	No-fascia-closure	Standardized difference
Cases	252	27	
Age, years, median (IQR)	70 (51–82)	71 (56–77)	0.034
Sex, male, *n* (%)	174 (69.0%)	21 (77.8%)	0.199
BMI, median (IQR)	22 (19–24)	25 (19–28)	0.499
Charlson Comorbidity index, median (IQR)	4 (1–6)	2 (1–4)	0.383
Past medical history, *n* (%)			
Laparotomy	55 (21.8%)	12 (44.4%)	0.495
Ventral hernia	1 (0.4%)	3 (11.1%)	0.473
Medication, *n* (%)			
Anticoagulants	21 (8.3%)	2 (7.4%)	0.034
Antiplatelets	38 (15.1%)	6 (22.2%)	0.184
Steroids	7 (2.8%)	1 (3.7%)	0.052
Etiology, *n* (%)			
Trauma	104 (41.3%)	8 (29.6%)	0.245
Nontraumatic hemorrhage	23 (9.1%)	3 (11.1%)	0.066
Abdominal compartment syndrome	11 (4.4%)	5 (18.5%)	0.456
Abdominal sepsis	62 (24.6%)	8 (29.6%)	0.113
Vital signs before surgery, median (IQR)			
SBP, mmHg	101 (82–121)	92 (80–119)	0.307
DBP, mmHg	60 (48–70)	58 (42–65)	0.210
Heart rate, /min	100 (88–120)	96 (70–110)	0.396
Respiratory rate, /min	24 (18–30)	20 (15–26)	0.909
SpO_2_, %	99 (96–100)	99 (95–100)	0.085
Ventilator use before surgery, *n* (%)	83 (32.9%)	15 (55.6%)	0.468
SOFA score before surgery, median (IQR)	6 (3–10)	11 (7–14)	0.386
Laboratory tests before surgery, median (IQR)			
Hemoglobin, g/dl	11.2 (9.2–13.0)	10.4 (8.7–12.7)	0.321
White blood cell, 10^3^/μl	11 (6–16)	13 (7–23)	0.336
Platelets, 10^3^/μl	168 (116–231)	135 (60–213)	0.477
PT-INR	1.2 (1.1–1.4)	1.3 (1.1–1.6)	0.283
Albumin, g/dl	2.9 (2.2–3.5)	2.6 (1.6–3.4)	0.366
Urea nitrogen, mg/dl	22 (15–37)	19 (13–39)	0.037
Creatinine, mg/dl	1.1 (0.9–1.8)	1.4 (0.9–2.3)	0.063
Resuscitation before surgery^a^, median (IQR)			
Fluid, ml	1000 (500–1950)	1250 (500–1560)	0.074
Red blood cell, ml	0 (0– 800)	0 (0– 800)	0.144
Fresh frozen plasma, ml	0 (0–240)	0 (0–480)	0.261
Platelet, U	0 (0–10)	10 (0–20)	0.315
Intraoperative information, median (IQR)			
Operation time, min	91 (51–147)	140 (48–194)	1.193
Fluid, ml	1920 (1120–3050)	1960 (530–3910)	0.090
Red blood cell, ml	800 (0– 1600)	1200 (400– 2800)	0.341
Fresh frozen plasma, ml	480 (0–1200)	960 (480–1920)	0.356
Platelet, U	0 (0–10)	10 (0–20)	0.467
Urine output, ml	100 (10–260)	110 (0–530)	0.413

DBP, diastolic blood pressure; IQR, interquartile range; PT-INR, prothrombin time - international normalized ratio; SBP, systolic blood pressure; SOFA, sequential organ failure assessment.

^a^ Resuscitation before surgery includes treatment within 6 h before surgery.

The frequency of use of each NPWT method (homemade, superficial NPWT kit, and open-abdomen kit) and the degree of negative pressure were similar between patients with and without primary fascia closure (Table 2). The median duration from the initiation of OA to primary fascia closure was 2 (1–4) days, and 2 (2–3) laparotomies were performed before primary fascia closure. Moreover, patients with primary fascia closure had a lower 28-day mortality and longer ICU-, ventilator-, and RRT-free days up to day 28, compared with those with no-fascia-closure. The incidence of most adverse events was fewer among patients with primary fascia closure, whereas more superficial SSIs were observed in those with primary fascia closure (Table 2).

**Table 2:** Temporary abdominal closure and postoperative information.

	Primary fascia closure	No-fascia-closure	Standardized difference
Method of NPWT, *n* (%)			
Homemade	136 (54.0%)	16 (59.3%)	0.107
Superficial NPWT kit	8 (3.2%)	2 (7.4%)	0.190
Open-abdomen kit	108 (42.9%)	9 (33.3%)	0.197
Degree of negative pressure, *n* (%)			
<50 mmHg	131 (53.5%)	13 (48.1%)	0.077
50–100 mmHg	17 (6.9%)	3 (11.1%)	0.154
>100 mmHg	97 (39.6%)	11 (40.7%)	0.046
Days to primary fascia closure, days, median (IQR)	2 (1–4)	–	–
Number of operations to primary fascia closure, median (IQR)	2 (2–3)	–	–
Mortality at 28 days, *n* (%)	32 (12.7%)	11 (40.7%)	**0.668**
ICU-free days to day 28, days, median (IQR)	15 (3–21)	0 (0–9)	**0.980**
Ventilator-free days to day 28, days, median (IQR)	18 (3–23)	0 (0–5)	**1.260**
RRT-free days to day 28, days, median (IQR)	28 (23–28)	0 (0–28)	**0.942**
Adverse events, *n* (%)			
Enteroatmospheric fistula	4 (1.2%)	4 (14.8%)	**0.519**
Anastomotic leakage	23 (9.1%)	5 (18.5%)	0.275
Wound dehiscence	16 (6.3%)	1 (3.7%)	0.121
Superficial SSI	67 (26.6%)	1 (3.7%)	**0.674**
Deep SSI	54 (21.5%)	10 (37.0%)	**0.348**
Ventral hernia	25 (10.1%)	–	–

Bold value indicates statistically significant.

IQR, interquartile range; NPWT, negative pressure wound therapy; RRT, renal replacement therapy; SSI, surgical site infection.

### Primary fascia closure and secondary outcomes

The association between the methods and the degree of negative pressure for NPWT and primary fascia closure is summarized in Table 3. In the primary GEE model that adjusted background risks for the failure of primary abdominal fascia closure, neither superficial NPWT kit nor open-abdomen kit use was associated with greater primary fascia closure achievement than homemade NPWT use [adjusted OR, 0.86 (95% CI: 0.10–7.40), *P*=0.894 and 5.66 (0.90–35.59), *P*=0.064, respectively]. Conversely, greater than 100 mmHg of negative pressure in NPWT was associated with fewer primary fascia closures [adjusted OR, 0.18 (95% CI: 0.50–0.69), *P*=0.012], whereas 50–100 mmHg was not [0.27 (0.05–1.37), *P*=0.114].

**Table 3:** NPWT and primary fascia closure.

	Primary fascia closure			Enteroatmospheric fistula		Adverse events^a^	
		OR (95% CI)	*P*		OR (95% CI)		OR (95% CI)
Method of NPWT, *n* (%)							
Homemade	136 (89.5%)	–	–	5 (3.3%)	–	72 (47.4%)	–
Superficial NPWT kit	8 (80.0%)	0.86 (0.10–7.40)	0.894	1 (10.0%)	2.21 (0.42–11.74)	3 (30.0%)	0.65 (0.78–1.69)
Open-abdomen kit	108 (92.3%)	5.66 (0.90–35.59)	0.064	1 (0.8%)	**0.02** **(0.00–0.50)**	46 (39.7%)	0.59 (0.28–1.22)
Degree of negative pressure, *n* (%)							
<50 mmHg	131 (91.0%)	–	–	5 (3.5%)	–	68 (47.2%)	–
50–100 mmHg	17 (85.0%)	0.27 (0.05–1.37)	0.114	0 (0.0%)	–	4 (20.0%)	**0.36** **(0.18–0.72)**
>100 mmHg	97 (89.8%)	**0.18** **(0.50–0.69)**	**0.012**	2 (1.8%)	**13.83** **(2.30–82.93)**	46 (43.0%)	1.33 (0.58–3.04)

Bold value indicates statistically significant.

^a^ Adverse events include enteroatmospheric fistula, anastomotic leakage, wound dehiscence, superficial/deep surgical site infection, and ventral hernia.

NPWT, negative-pressure wound therapy; OR, odds ratio.

The sensitivity analyses using all relevant covariates similarly revealed the relationship between greater than 100 mmHg of negative pressure in NPWT and a lower rate of primary fascia closure [OR, 0.25 (0.07–0.93); Table S1, Supplemental Digital Content 2, http://links.lww.com/JS9/A945], but no association between the methods of NPWT and primary fascia closure. In addition, other two GEE models that were developed to balance the background adjustment and overfitting of the model, and the multivariate logistic regression model, found that greater than 100 mmHg of negative pressure was related to fewer primary fascia closures [OR, 0.23 (0.08–0.64), 0.007 (0.01–0.44), and 0.10 (0.01–0.78), respectively; Table S1, Supplemental Digital Content 2, http://links.lww.com/JS9/A945].

A lower incidence of enteroatmospheric fistula was associated with the use of the open-abdomen kit compared with using homemade NPWT [adjusted OR, 0.02 (95% CI: 0.00–0.50); Table 3], whereas greater than 100 mmHg of negative pressure in NPWT during tentative abdominal closure was significantly associated with a higher incidence of enteroatmospheric fistula [adjusted OR, 13.83 (95% CI: 2.30–82.93); Table 3]. Moreover, while the methods of NPWT were not related to the incidence of all adverse events, 50–100 mmHg of negative pressure was associated with fewer adverse events [adjusted OR, 0.36 (95% CI: 0.18–0.72)].

### Subgroup analysis

Subgroup analyses (Table 4) revealed a relationship between failure of primary fascia closure and a higher degree of negative pressure (>100 mmHg) in several subgroups, namely, patients who underwent OA after 2017, those with a limited amount of intra-abdominal bleeding (≤500 ml), and those with lower postoperative lactate (<4 mmol/l). Conversely, patients with a higher postoperative lactate (≥4 mmol/l) had a comparable rate of primary fascia closure regardless of the degree of negative pressure in NPWT.

**Table 4:** Subgroup analysis of primary fascia closure.

	Primary fascia closure
	OR (95% CI)
In recent years (after 2017)	
Method of NPWT	
Homemade	—
Superficial NPWT kit	3.55 (0.23–55.26)
Open-abdomen kit	**23.90** **(1.83–312.00)**
Degree of negative pressure, *n* (%)	
<50 mmHg	—
50–100 mmHg	0.07 (0.00–1.11)
>100 mmHg	**0.04** **(0.00–0.40)**
Intraoperative hemorrhage ≤500 ml	
Method of NPWT	
Homemade	—
Superficial NPWT kit	—^a^
Open-abdomen kit	**6.28** **(1.85–21.28)**
Degree of negative pressure, *n* (%)	
<50 mmHg	–
50–100 mmHg	**0.15** **(0.06–0.34)**
>100 mmHg	**0.99** **(0.03–0.99)**
Intraoperative hemorrhage ≤1000 ml	
Method of NPWT	
Homemade	—
Superficial NPWT kit	–^a^
Open-abdomen kit	4.87 (0.63–37.56)
Degree of negative pressure, *n* (%)	
<50 mmHg	–
50–100 mmHg	**0.27** **(0.10–0.77)**
>100 mmHg	0.53 (0.03–8.52)
Postoperative lactate ≥ 4 mmol/l	
Method of NPWT	
Homemade	—
Superficial NPWT kit	—^a^
Open-abdomen kit	2.85 (0.28–28.65)
Degree of negative pressure, *n* (%)	
<50 mmHg	—
50–100 mmHg	0.15 (0.01–1.57)
>100 mmHg	0.42 (0.05–3.61)
Postoperative lactate <4 mmol/l	
Method of NPWT	
Homemade	—
Superficial NPWT kit	—^a^
Open-abdomen kit	—^a^
Degree of negative pressure, *n* (%)	
<50 mmHg	—
50–100 mmHg	—^a^
>100 mmHg	**0.11** **(0.02–0.58)**

Bold value indicates statistically significant.

NPWT, negative-pressure wound therapy; OR, odds ratio.

^a^ OR could not be calculated in the model due to the limited number of cases with no primary fascia closure.

Furthermore, the moderate degree of negative pressure (50–100 mmHg) was also associated with unsuccessful primary fascia closure among patients with less than or equal to 1000 ml of intra-abdominal bleeding. Regarding the methods of NPWT, the use of an open-abdomen kit was associated with a higher rate of primary fascia closure in patients in recent years and those with less than or equal to 500 ml of intra-abdominal bleeding.

## Discussion

This study revealed that greater than 100 mmHg of negative pressure in NPWT was associated with failure of primary abdominal fascia closure, compared with less than 50 mmHg of negative pressure, in patients who underwent OA. Conversely, the use of the superficial NPWT kit or open-abdomen kit was not associated with a greater achievement of primary fascia closure, than the use of homemade NPWT, in the current study.

Possible pathophysiological mechanisms behind the relationship between greater than 100 mmHg of negative pressure and unsuccessful primary fascia closure would include a higher incidence of enteroatmospheric fistula due to higher negative pressure during OA^25,26^. A systematic review of OA methods reported that the higher negative pressure might damage organs^9^, although the appropriate negative pressure could not be determined due to insufficient data. In addition, an anastomosis, dilated bowel, or ischemic intestine would be vulnerable to nonphysiological negative pressure^27,28^, which is common in patients who required OA. Furthermore, an animal study indicated reducing mesenteric blood flow with increasing intra-abdominal negative pressure during OA^29^, suggesting that even a healthy intestine would be at risk of perforation under higher negative pressure. Notably, enteroatmospheric fistula was more frequent in patients with greater than 100 mmHg of negative pressure than in those with less than 50 mmHg in the current study.

While 50–100 mmHg of negative pressure was not associated with the success rate of primary fascia closure, it was related to fewer adverse events than less than 50 mmHg. A reason might be the effective removal of fluid, which could decrease the incidence of deep SSI^30,31^. Therefore, the results in this study suggest 50–100 mmHg would be an optimal target for the level of negative pressure for tentative abdominal closure. However, patients with limited intra-abdominal bleeding had fewer primary fascia closures even when the negative pressure was 50–100 mmHg. The benefits of fluid removal under higher negative pressure in this population would be outweighed by the risks of enteroatmospheric fistula, and less than 50 mmHg would be recommended.

Although no method of NPWT was associated with the achievement of primary fascia closure in this study, patients treated with the open-abdomen kit experienced a lower incidence of enteroatmospheric fistula, compared with those with homemade NPWT. As most commercially available open-abdomen kits are designed to apply less direct pressure to the bowel surface^32^, they would protect the hollow viscera from nonphysiological negative pressure. Notably, the use of the open-abdomen kit was associated with successful primary fascia closure in the subgroup of patients who underwent OA in recent years.

Importantly, unfavorable outcomes due to high negative pressure were observed only in patients with a lower postoperative lactate (<4 mmol/l) and those with limited intra-abdominal bleeding (≤500 ml). Therefore, the optimal method or degree of negative pressure for tentative abdominal closure would need to be tailored in hemodynamically unstable patients with massive intra-abdominal hemorrhage. However, the results of the subgroup analyses should be interpreted with caution, because of the limited sample size.

Based on the current results, less than 100 mmHg of negative pressure in NPWT probably should be avoided unless such a high degree of negative pressure is required for suctioning massive intra-abdominal fluid. In addition, an open-abdomen kit may be useful to avoid enteroatmospheric fistula. To validate these optimal NPWTs for tentative abdominal closure in OA, prospective study with a large sample size should be conducted.

Various academic societies recognize that patients treated with OA are heterogeneous and high-quality clinical studies to clarify indications, management, and definitive closure of OA have been limited. Therefore, they support the International Register of Open Abdomen, that was initiated in 2015 and has reported various novel data regarding OA^4,5,10,22,23^. While several clinical factors have been well examined among patients with OA, including age, race, obesity, and intraperitoneal fluid instillation, the optimal NPWT has not been elucidated yet. The current results should be further examined using such a large cohort in the near future.

## Limitations

This study’s findings must be interpreted in the context of its design. We retrospectively retrieved data from medical charts and could not obtain indications for each method of NPWT and the degree of negative pressure. Therefore, our results might have differed if the reasons for using specific NPWT methods depended on strong but unrecorded prognostic factors, such as the quality of prehospital care and the skills and experience of the surgeons. Another limitation was the lack of detailed clinical information for the development of enteroatmospheric fistula. Although supraphysiological intra-abdominal negative pressure could cause bowel damage, it cannot be objectively evaluated. Furthermore, the current study excluded patients who died within 48 h of the initiation of OA. As some critically ill patients might need a higher negative pressure to achieve hemorrhage packing or remove massive intra-abdominal fluid collection, the generalizability of the results for such a population is limited. Finally, considering slight changes in the pressure of NPWT would exist even in the same patients, the negative pressure was analyzed based on 50 mmHg increments. Other optimal ranges for negative pressure in NPWT for tentative abdominal closure may exist depending on the indication, timing, and duration of OA.

## Conclusion

This study revealed that a higher degree of negative pressure (>100 mmHg) was associated with unsuccessful primary abdominal fascia closure and more frequent enteroatmospheric fistulae. While the use of an open-abdomen kit with 50–100 mmHg of negative pressure is recommended as an optimal method for tentative abdominal closure for OA, this should be validated by further studies.

## Ethical approval

All collaborating hospitals obtained individual local institutional review board approval for conducting research with human subjects before study initiation. This study was approved by the Institutional Review Board at Keio University School of Medicine, Tokyo, Japan on 29 March 2021 (approval no. 20200338).

## Consent

Informed consent was waived because of the anonymous nature of the data.

## Sources of funding

This study received a research grant from the Japanese Society for Abdominal Emergency Medicine (2020-P-05).

## Author contribution

R.Y., M.S., H.S., K.S., and J.S.: designed the study; R.Y., S.K., M.S., H.S., T.M., Y.S., K.S., Y.K., R.K., K.K., T.K., S.Y., and S.W.: performed data collection; J.S.: managed the quality control; R.Y. and S.W.: performed data analysis, interpretation, writing, and critical revision. All authors have revised the article.

## Conflicts of interest disclosure

The authors declare that they have no conflicts of interest.

## Research registration unique identifying number (UIN)

The study was registered at the University Hospital Medical Information Network Clinical Trial Registry on 23 June 2021 (UMIN-CTR ID, UMIN000044619) prior to study initiation, which is available at https://center6.umin.ac.jp/cgi-open-bin/ctr/ctr_view.cgi?recptno=R000050757.

## Guarantor

Ryo Yamamoto, MD, PhD, Department of Emergency and Critical Care Medicine, Keio University School of Medicine, Tokyo 160-8582, Japan. E-mail: ryo.yamamoto@gmail.com.

## Provenance and peer review

This manuscript was not invited.

## Data availability statement

The data of this study are available from the Japanese Society for Abdominal Emergency Medicine; however, restrictions apply to the data availability, which were used under license for this study and so are not publicly available. However, data are available from the authors upon reasonable request with permission from the Japanese Society for Abdominal Emergency Medicine.

## Supplementary Material

SUPPLEMENTARY MATERIAL
