# Supplementary material for: Optimal tentative abdominal closure for open abdomen: a multicenter retrospective observational study (OPTITAC study)
Source: Int J Surg. 2023 Sep 2;109(12):4049–56. doi: 10.1097/JS9.0000000000000687 (PMC10720862; doi:10.1097/JS9.0000000000000687)
Supplement: SUPPLEMENTARY MATERIAL [file js9-109-4049-s002.docx]

| Table S1. NPWT and primary fascia closure in sensitivity analysis | | | | | |  |
| --- | --- | --- | --- | --- | --- | --- |
|  |  |  | Primary fascia closure | | |  |
|  |  |  | OR (95% CI) | | p value |  |
| With all pre-operative covariates | | |  |  |  |  |
|  | Method of NPWT | |  |  |  |  |
|  |  | Home-made | - | | - |  |
|  |  | Superficial NPWT kit | 0.44 | (0.02–9.83) | 0.608 |  |
|  |  | Open-abdomen kit | 3.42 | (0.64–18.21) | 0.194 |  |
|  | Degree of negative pressure, n(%) | |  |  |  |  |
|  |  | < 50 mmHg | - | | - |  |
|  |  | 50 - 100 mmHg | 0.17 | (0.04–0.63) | 0.009 |  |
|  |  | > 100 mmHg | 0.25 | (0.07–0.93) | 0.039 |  |
| With demographics and preoperative hemodynamics | | |  |  |  |  |
|  | Method of NPWT | |  |  |  |  |
|  |  | Home-made | - | | - |  |
|  |  | Superficial NPWT kit | 0.53 | (0.06–4.88) | 0.577 |  |
|  |  | Open-abdomen kit | 4.69 | (1.15–19.18) | 0.032 |  |
|  | Degree of negative pressure, n(%) | |  |  |  |  |
|  |  | < 50 mmHg | - | | - |  |
|  |  | 50 - 100 mmHg | 0.29 | (0.10–0.81) | 0.018 |  |
|  |  | > 100 mmHg | 0.23 | (0.08–0.64) | 0.005 |  |
| With postoperative information | | |  |  |  |  |
|  | Method of NPWT | |  |  |  |  |
|  |  | Home-made | - | | - |  |
|  |  | Superficial NPWT kit | 1.06 | (0.99–7.16) | 0.953 |  |
|  |  | Open-abdomen kit | 14.25 | (1.35–150.81) | 0.027 |  |
|  | Degree of negative pressure, n(%) | |  |  |  |  |
|  |  | < 50 mmHg | - | | - |  |
|  |  | 50 - 100 mmHg | 0.10 | (0.01–1.69) | 0.112 |  |
|  |  | > 100 mmHg | 0.07 | (0.01–0.44) | 0.005 |  |
| Multivariate logistic regression* | | |  |  |  |  |
|  | Method of NPWT | |  |  |  |  |
|  |  | Home-made | - | | - |  |
|  |  | Superficial NPWT kit | 1.35 | (0.11–16.97) | 0.817 |  |
|  |  | Open-abdomen kit | 8.92 | (1.10–72.15) | 0.040 |  |
|  | Degree of negative pressure, n(%) | |  |  |  |  |
|  |  | < 50 mmHg | - | | - |  |
|  |  | 50 - 100 mmHg | 0.17 | (0.02–1.29) | 0.087 |  |
|  |  | > 100 mmHg | 0.10 | (0.01–0.78) | 0.027 |  |
| NPWT = negative pressure wound therapy and OR = odds ratio. *Logistic regression analysis was conducted with non-imputed data. | | | | | |  |
|  |  |  |  |  |  |  |
